# Managing Congenital Lobar Overinflation Associated with Congenital Heart Disease

**DOI:** 10.3390/children7090113

**Published:** 2020-08-28

**Authors:** Ranjit I. Kylat

**Affiliations:** Department of Pediatrics, University of Arizona, 1501 N Campbell Ave, Tucson, AZ 85724, USA; rkylat@gmail.com

**Keywords:** congenital lobar overinflation, congenital lobar emphysema, congenital heart defects, bronchoscopy, lobectomy, cardiac surgical procedures

## Abstract

The incidence of congenital lobar overinflation (CLO) is reported at 1 in 20,000–30,000 live births and represents 10% of all congenital lung malformations. The occurrence of concomitant congenital heart disease (CHD) and CLO ranges from 12% to 20%. There are diverging views in the management as to whether early lobectomy or repair of the cardiac defect, with the assumption that respiratory symptomatology would gradually resolve, or a combined lung and cardiac repair would be the ideal first step in the management. In concomitant CLO and CHD, the surgical decision has to be individualized. Prior to surgical intervention a thorough evaluation may be needed with contrast computed tomography (CT) or magnetic resonance imaging (MRI), bronchoscopy, and if needed cardiac catheterization. CLO improves with management of many left to right shunts and in those with anomalous vessels, but early lobectomy or combined approach may be considered in those symptomatic patients with more complex CHD.

## 1. Introduction

Congenital lobar overinflation (CLO) is an uncommon anomaly and represents 10% of all congenital lung malformations [1,2]. The occurrence is reported at 1 in 20,000–30,000 live births but may be under reported [3]. CLO can present as respiratory distress in the neonate or during infancy but can also be asymptomatic [1,2]. The occurrence of concomitant congenital heart disease (CHD) and CLO is reported to be between 12% and 20% [4]. There is dearth of clear evidence as to the optimal management in affected infants with respiratory symptoms. There are diverging management views as to whether early lobectomy or repair of the cardiac defect, with the assumption that respiratory symptomatology would gradually resolve, or a combined lung and cardiac repair would be the ideal first step. Herein, the conflicting views on the appropriate initial surgical approach are illustrated.

## 2. Case

**Figure 1:**
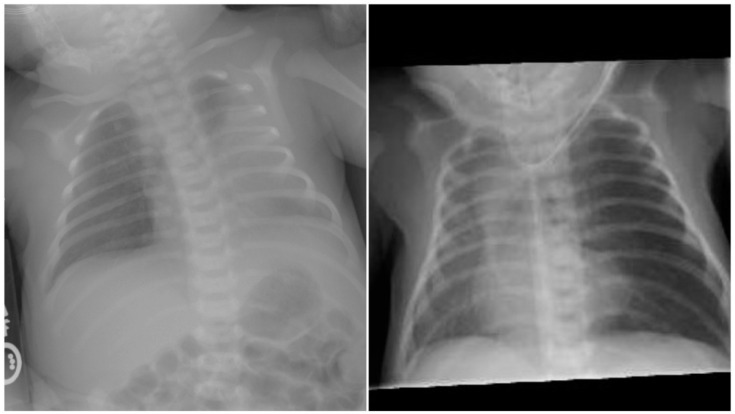
Left panel depicts anterio-posterior chest radiograph on the first day with left-sided opacification. Right panel reveals radiograph at 7 days with hyperinflation of the left and mediastinal shift.

**Figure 2:**
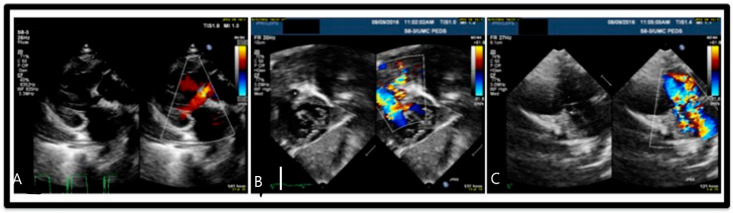
Echocardiogram: (**A**) Long axis view of ventricular septal defect (VSD) demonstrating pulmonary vessel stenosis. (**B**) Subcostal four chamber view of VSD with aorta overriding pulmonary artery and feeding into right ventricle. (**C**) View of double outlet pulmonary artery and aorta from right ventricle with VSD present.

**Figure 3:**
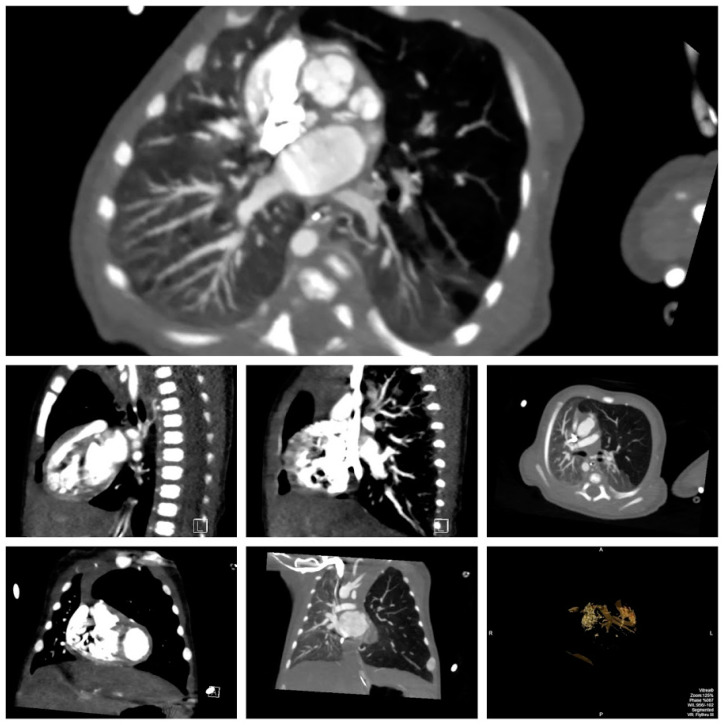
Multiple views on cardiac computed tomography (CT) demonstrating tertiary tracheal bronchus supplying the lingula with stenosis at the origin, double outlet right ventricle (DORV) with transposition of the great vessels, hyperinflated left upper lobe, partially atelectatic left upper and lower lobes, herniation to the right, and 3D reconstruction of airway.

A 21-year-old primigravida with a prenatal diagnosis of a fetus with cardiac anomalies delivered a male infant at 37 weeks. The newborn infant at birth was asymptomatic with normal oxygen saturation. Initial studies demonstrated left upper lobe infiltrate that resolved by the second day (Figure 1 left). The patient was found to have double outlet right ventricle (DORV), with mitral valve hypoplasia and stenosis due to supramitral ring, ventricular septal defect (VSD), hypoplastic pulmonic valve with stenosis, malposed great vessels (aorta rightward and anterior) and left pulmonary artery (LPA) anomaly (Figure 2). The patient developed respiratory symptoms initially attributed to the underlying cardiac diagnosis, but over the next few weeks evolved with left upper lobe hyperinflation, mediastinal shift, and segmental atelectasis (Figure 1 right). On cardiac computed tomography (CT), a left lingular CLO along with a stenotic tracheal bronchus supplying the lingula, and an anomalous course of the LPA (Figure 3). Bronchoscopy confirmed the stenotic tracheal bronchus on the left. Due to worsening respiratory distress and the patient requiring respiratory support, at six weeks of age, the left upper lobe was resected with resolution of symptoms. Two months later, he underwent a successful DORV repair, which included supramitral ring resection with resection of right ventricular muscle bundles, enlargement of the VSD, patch baffle (Gore-Tex, W.L. Gore & associates, Flagstaff, AZ, USA) of the VSD to the rightward aorta, infundibular patch augmentation, and LPA plasty (spatulated anastomosis between the proximal ascending LPA and distal descending LPA). At the 18 months patient follow-up, he did not exhibit significant symptomatology and had normal growth.

## 3. Discussion

CLO is also known as congenital lobar emphysema, congenital alveolar over-distension, and infantile lobar emphysema. It is a developmental anomaly of the respiratory tract, which results in hyperinflation of one or more lobes of the lung. Generally, 50% of CLO cases are classified as idiopathic and the rest are either of intrinsic or extrinsic pulmonary etiologies. Intrinsic causes of CLO include bronchial structural abnormalities or developmental deficiencies in bronchial cartilage, and extrinsic etiologies include bronchial compression such as cardiopulmonary vascular anomalies, intrathoracic masses, rings, and slings. Early in pregnancy, CLO can grow secondary to fluid trapping, but most of these will regress and can be indistinguishable from normal lung tissue on an ultrasound scan (US). Males have a 2 to 3 times higher predilection as compared to females. Prenatal fetal ultrasound detects only 25% of cases [1]. Fetal magnetic resonance imaging (MRI) can distinguish CLO from other lung malformations. The left upper lobe is the most common lobe affected followed by right middle and right upper [1]. In the idiopathic type, transient extrinsic compression of the left upper lobe bronchus by the ductus arteriosus in utero is hypothesized to be the reason for it being the commonest location.

In 12–14% of patients, CLO has been shown to be associated with congenital heart disease (CHD), with 50% being ventricular septal defects (VSD) [4,5]. As CLO symptoms present similarly to those of CHD, often diagnosis is not made until after cardiac repair. The appropriate management decision in this setting poses challenging questions with many factors to be taken into account [4,5]. Resolution of right middle lobe emphysema was observed both in spontaneous and operative correction of left to right shunts [6,7]. In these reports, patients have sometimes had improvement of CLO from cardiac repair, suggesting lobectomy should not be the standard management in the left to right shunt cases of CHD. It has been well documented that in certain cardiac defects especially the left to right shunts, reversible airway obstruction could be due to anatomic compression from anomalous or dilated vessels as in the absent pulmonary valve, rings, and pulmonary slings [8]. Therein, the resolution of CLO is attributed to relief of obstruction when there is normalization of the dilated vessels. In contrast, in a report on three infants with severe respiratory symptoms attributed to VSD, surgery for the associated CLO led to complete resolution of symptomatology, and cardiac surgery was not required [5]. Additionally, in patients with known CLO and CHD, successful combined pulmonary and cardiac surgery has been reported, thereby eliminating a separate surgical procedure and allowing concomitant healing and growth of both heart and lung [9]. However, optimizing pulmonary function, prior to cardiac surgery, potentially prevents prolonged mechanical ventilation requirement and improves postoperative outcomes from delayed repair. This option for management, nonetheless, requires time between surgeries for recovery to occur. In certain CLO situations, such as in an anomalous pulmonary artery, it would be clear that the vascular repair would be the only treatment needed [10]. The resolution of emphysematous lung after cardiac repair in some cases of left to right shunting is possibly due to the vascular compression and airway malacia that leads to air trapping, and it is well known that all cases of air trapping should not be called CLO. The air trapping in central airway vascular compression associated with CHD such as absent pulmonary valve syndrome and vascular rings and slings should be differentiated from CLO, and if there is a dilemma, then delaying lobectomy until after 1 year of age gives a more reliable indication for the need for resection. This would limit the resection of a normal lung, allowing it to expand and eventually compensate.

The etiologic mechanisms for CLO are unusual flaccidity of the bronchial wall; abnormal or malformed bronchial cartilage; poorly developed cartilaginous rings (infantile bronchus); pulmonary artery sling; pulmonary rotation anomaly; bronchogenic cyst duplication of esophagus; mediastinal mass; polyalveolar lobe; bronchial polyp; redundant bronchial mucosal folds, septae, and webs; or compression of the bronchus by an abnormal vessel, collateral, or by dilated pulmonary arteries or an enlarged left atrium [11]. The characteristic finding is a progressive pulmonary hyperinflation of certain segments or one or more lobes, which is the result of a one-way valve (“ball-valve”) mechanism of the abnormal airway causing air trapping. This does not usually result in destruction of the alveolar walls but can lead to compression atelectasis of the rest of the segments or lobes leading to mediastinal shift, increased intrathoracic pressure, diminished respiratory reserve, and ventilation–perfusion mismatch, resulting in hypoxia. In many of these conditions, the common pathway is a result of a disruption in the bronchopulmonary development with abnormal embryonic endodermal and mesodermal components of the lung.

Many CLO patients can be asymptomatic or have only mild respiratory distress at birth but could have progression of symptoms during infancy. The diagnosis is usually made radiologically with initial opacifications from retained amniotic fluid subsequently evolving into hyperinflation and herniation. If asymptomatic or if the symptoms are mild, the management is conservative. If the patient has clinical progression or is clinically severe, the treatment recommended is a lobectomy [11].

In concomitant CLO and CHD, the surgical decision has to be individualized. In these nstances, the common cardiac anomalies associated include VSD, patent ductus arteriosus, interruption of the aortic arch, and tetralogy of Fallot with absent pulmonary valve, and as the clinical scenarios are variable, tailoring an individual management pathway is ideal. The factors to be considered would be the type and severity of cardiac pathology, the presence and degree of cardiac failure, presence of significant atelectasis of the adjacent lung size of pulmonary vessels, severity of pulmonary arterial hypertension, presence of anomalous vessels, collaterals or ductus arteriosus, and above all the need for mechanical ventilation (with its propensity to worsen CLO). Most of the infants would need bronchoscopy and contrast cardiac and chest imaging to evaluate the degree of bronchial anomaly, presence of any accessory vessels, extrinsic compression, and other anomalies. Cardiac catheterization, bronchography, and nuclear medicine lung scans are not usually required. In left to right shunts, if the respiratory symptomatology is incongruous to the degree of cardiac failure by noninvasive or laboratory tests, it would be reasonable to assume that the CLO does play a more significant role when compared to the cardiac lesion [6,7]. However, it does not automatically mean that lobectomy would be the initial treatment of choice, as many of these types of CLO are totally reversible. If appropriate, aggressive medical management or interventional methods of managing left to right shunt or anomalous vessels would be ideal in such scenarios. Although CLE improves with management of many left to right shunts and in those with anomalous vessels, early lobectomy or combined approach may be considered in those symptomatic patients with more complex CHD. There would be instances where the patient requiring surgical repair would have difficulty weaning off the ventilator when lobectomy would be required, especially if the emphysematous lung does not allow the re-expansion of atelectatic areas in the postoperative period. A combined lobectomy and cardiac repair would generally be feasible only if the type of cardiac repair is of moderate complexity.

In the patient described above, the decision to perform CLO repair was based on progressive respiratory deterioration with the need for mechanical ventilation, complex cardiac surgery required, and therefore optimal pulmonary function needed for intraoperative stability, postoperative recovery, and likelihood of worsening of emphysema if mechanical ventilation was needed. In addition, the presence of the CLO in the left upper lobe, its detection soon after birth, and the stenotic bronchus suggested an irreversible pathology and the cardiac pathology and type of repair permitted the option of waiting 4 to 6 weeks after lobectomy. Thereby, delaying the cardiac repair resulted in a favorable intraoperative and postoperative outcome and early discharge of the patient. The association of DORV and CLO with involvement of the lingula and the presence of tertiary tracheal bronchus demonstrates a unique presentation. Multidetector-row computed tomography can provide accurate detection of segmental or lobar emphysema in CHD with increased pulmonary blood flow [12]. Compensatory lung development has been well described [13].

## 4. Conclusions

In concomitant CLO and CHD, the surgical decision has to be individualized. Prior to surgical intervention a thorough evaluation may be needed with contrast CT or MRI, bronchoscopy, and if needed, cardiac catheterization. CLO improves with management of many left to right shunts and in those with anomalous vessels, but early lobectomy or combined approach may be considered in those symptomatic patients with more complex CHD.
